# FolX from *Pseudomonas aeruginosa* is octameric in both crystal and solution

**DOI:** 10.1016/j.febslet.2012.03.031

**Published:** 2012-04-24

**Authors:** Mads Gabrielsen, Katherine S.H. Beckham, Richard J. Cogdell, Olwyn Byron, Andrew J. Roe

**Affiliations:** a Institute of Infection, Immunity and Immunology, College of Medical, Veterinary and Life Sciences, Sir Graeme Davies Building, University of Glasgow, Glasgow G12 8QQ, UK; b Institute of Molecular, Cell and Systems Biology, College of Medical, Veterinary and Life Sciences, Sir Graeme Davies Building, University of Glasgow, Glasgow G12 8QQ, UK; c School of Life Sciences, College of Medical, Veterinary and Life Sciences, Sir Graeme Davies Building, University of Glasgow, Glasgow G12 8QQ, UK

**Keywords:** FolX, 7,8-dihydroneopterin-triphosphate-epimerase, AUC, analytical ultracentrifugation, SAXS, small-angle X-ray scattering, SV, sedimentation velocity, SE, sedimentation equilibrium, Pteridine biosynthesis, X-ray structure, Solution structure

## Abstract

FolX encodes an epimerase that forms one step of the tetrahydrofolate biosynthetic pathway, which is of interest as it is an established target for important drugs. Here we report the crystal structure of FolX from the bacterial opportunistic pathogen *Pseudomonas aeruginosa*, as well as a detailed analysis of the protein in solution, using analytical ultracentrifugation (AUC) and small-angle X-ray scattering (SAXS). In combination, these techniques confirm that the protein is an octamer both in the crystal structure, and in solution.

**Structured summary of protein interactions:**

**FolX** and **FolX**bind by x-ray crystallography (View interaction)

**FolX** and **FolX**bind by cosedimentation in solution (View interaction)

**FolX** and **FolX**bind by x ray scattering (View interaction)

## Highlights

► Structural investigation of FolX from *Pseudomonas aeruginosa*. ► A combination of AUC and SAXS allows the X-ray structure to be ‘improved’. ► FolX is shown an octamer in both crystal and in solution. ► A network of 16 hydrogen bonds and 8 salt bridges provide octamer stability.

## 1 Introduction

**Fig. 1:**
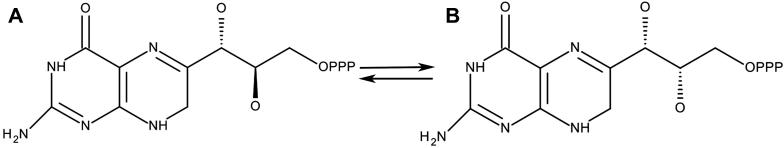
Chemdraw representation of the epimerase reaction catalysed by FolX, where dihydroneopterin is converted to dihydromonapterin triphosphate.

The tetrahydrofolate biosynthetic pathway is an established target for important antibiotics including trimethoprim that inhibits the activity of bacterial dihydrofolate reductase [1–3]. However, with the ever-increasing spread of antibiotic resistance in bacteria, there is an urgent need to explore alternative approaches to controlling pathogens. Recent work has elucidated the target proteins of one group of so-called “anti-virulence” compounds, the salicylidene acylhydrazides. In this published study [4], several target proteins were identified including FolX, encoded by a gene only carried by the gammaproteobacteria. FolX has been shown to act as an epimerase (Fig. 1) in conjunction with FolM, a reductase, during the conversion of dihydroneopterin triphosphate to tetrahydrobiopterin [5]. Therefore, FolX and FolM are essential for tetrahydromonapterin synthesis in species such as *Escherichia coli* and *Pseudomonas aeruginosa*.

Here we report the crystal structure of FolX from *P. aeruginosa* as well as a detailed analysis of the protein in solution using analytical ultracentrifugation (AUC) and small-angle X-ray scattering (SAXS). The combination of these techniques confirms that the octameric crystal structure is consistent with the biological state of FolX in solution.

## 2 Materials and methods

### 2.1 Protein expression and purification

The gene encoding FolX was amplified from *P. aeruginosa* genomic DNA and cloned into the vector pET-151 (Invitrogen) [4]. The expression and purification was performed following previously published methods [6].

### 2.2 Crystallisation

Purified FolX was dialysed against 20 mM Tris pH 7.5, 50 mM NaCl and concentrated to approximately 6 mg ml^−1^, based on the absorbance at 280 nm (A_280_) and an extinction coefficient of 5960 M cm^−1^ derived from the sequence composition. Screens were set up using commercially available crystallisation kits, using vapour diffusion, with drops consisting of 500 nl protein solution and 500 nl reservoir. Cubes (0.2 × 0.2 × 0.2 mm) appeared in conditions, containing 40% (v/v) 1,2-propanediol, 100 mM HEPES pH 7.5, within a week, at room temperature.

### 2.3 Data collection, processing and structure solution

Crystals were flash-frozen in liquid nitrogen, with no further cryo-protection, and brought to Diamond Light Source, station I03. Data were collected on a PILATES 6M detector, at a wavelength of 0.97625 Å. A total of 127° of data were collected using an increment of 0.15°. Data were processed using MOSFLM [7] and scaled in SCALA [8]. The structure was determined using the Balbes molecular replacement server [9], which identified PDB entry 1B9L [10] as the best search model. The solved structure was refined with BUSTER [11], using TLS parameterisation and torsion restraints from the search model, and inspected and altered when required, using COOT [12]. Waters were added using BUSTER. The geometry of the structure was validated by MOLPROBITY [13]. The data showed signs of anisotropy and were processed to 3.0 Å, based on the statistical factors presented in Table 1, although diffraction spots could be detected closer to 2.5 Å.

### 2.4 Analytical ultracentrifugation

Purified FolX was dialysed against 20 mM Tris pH 7.5, 150 mM NaCl and concentrated to approximately 10 mg ml^−1^, based on the A_280_. Analytical ultracentrifugation (AUC) was carried out in a Beckman Coulter (Palo Alto, CA) Optima XL-I analytical ultracentrifuge. Sedimentation velocity (SV) experiments were performed at 4 °C at a rotor speed of 49 krpm. 360 μl of sample, at concentrations of FolX ranging between 0.2 and 10 mg ml^−1^, were loaded into double sector centrepieces. Data were acquired every 7 min with interference and absorbance optics and were subsequently analysed using SEDFIT [14]. The partial specific volume of FolX (0.737/0.743 g ml^−1^), the buffer density (1.00677/1.00499 g ml^−1^) and viscosity (0.0156/0.0102 P) at 4 °C and 20 °C respectively, were all calculated using the program SEDNTERP [15]. Sedimentation equilibrium (SE) experiments were carried out with the same range of FolX concentrations using 90 μl of sample with a rotor speed of 23 krpm. Scans were taken every 3 h until analysis of the scans, using WinMATCH (Jeffrey Lary, University of Connecticut, Storrs, CT, USA), indicated that equilibrium had been reached. SE data were analysed using Origin and were fitted with a tetramer–octamer model with non-ideality. The dissociation constant was calculated using the method described by Solovyova *et al.* [16].

### 2.5 Small angle X-ray scattering measurements

SAXS data were collected at the ESRF ID14 EH3 beamline for samples with a range of FolX concentrations of between 0.5 and 6 mg ml^−1^ in 20 mM Tris pH 7.5, 150 mM NaCl. No concentration dependence effects were observed, therefore data from the highest concentration (6 mg ml^−1^) were processed for further analysis. Initial processing of the data was done using PRIMUS and p(r) analysis was carried out using GNOM [17]. The resolution of the data was calculated from the highest angle at which useable scattering data were recorded [16] *Ab initio* models of FolX were generated from the experimental data using DAMMIF [18]. Twenty DAMMIF models were superimposed and averaged using DAMAVER [19] and the averaged model was superimposed onto the crystal structure using SUPCOMB [20].

Figures were made using ALINE [21] and PyMOL [22].

## 3 Results and discussion

### 3.1 Monomeric structure

**Fig. 2:**
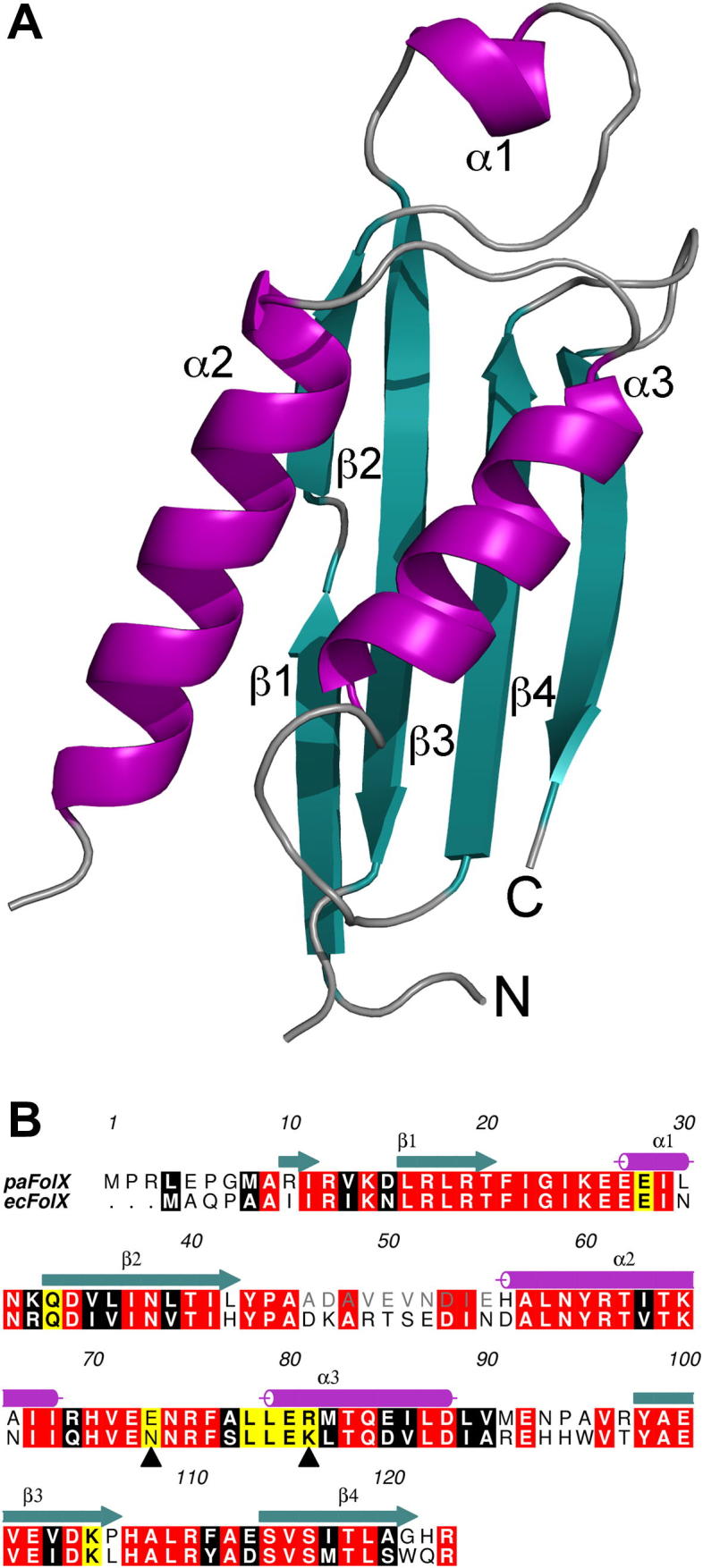
(A) Cartoon representation of a subunit of FolX. The helices are coloured in purple and the strands are coloured in teal. The N- and C-termini have been labelled. (B) Structural alignment of FolX from *P. aeruginosa* and *E. coli*, with secondary structure elements coloured as above. Highlighted in yellow are the residues involved in the putative active site. Residues in grey indicate residues not modelled in the electron density.

The monomeric structure of FolX comprised a four-stranded antiparallel sheet, composed of β1 (residues 10–12 and 16–20), β2 (residues 33–42), β3 (residues 98–106) and β4 (residues 114–121) (Fig. 2A). The broken β1 strand, caused by a small kink introduced in the strand, was also observed in the structure of FolX from *E. coli* [10]. A short α-helix (α1, 4 residues) is located in the loop between sheets β1 and β2, whereas α -helices α2 and α3 are nestled against the sheet on the concave side. The structure is not complete, as only residues 6–46 and 55–122 can be observed in the electron density. This disordered region exhibits the greatest sequence disparity compared with *E. coli* FolX (Fig. 2B) although it is located opposite to the active site and is therefore unlikely to affect overall function. There are a number of side chains that cannot be observed in the electron density, and have, accordingly, been cut back to the last ordered atom.

The sequence and structure of FolX are highly conserved throughout the gammaproteobacteria, and the sequence identity and similarity between FolX from *P. aeruginosa* and *E. coli* is 60% and 78% respectively (based on a level of 0.7, using the ALSCRIPT algorithm [23]) (Fig. 2B). The structures of the two homologues superpose with a root-mean square deviation (r.m.s.d.) of 0.99 Å for 100 Cα_._. The main differences are mostly limited to the loop between α2 and β3. These differences are not localised near the interfaces, or the putative active site.

### 3.2 Quaternary structure

**Fig. 3:**
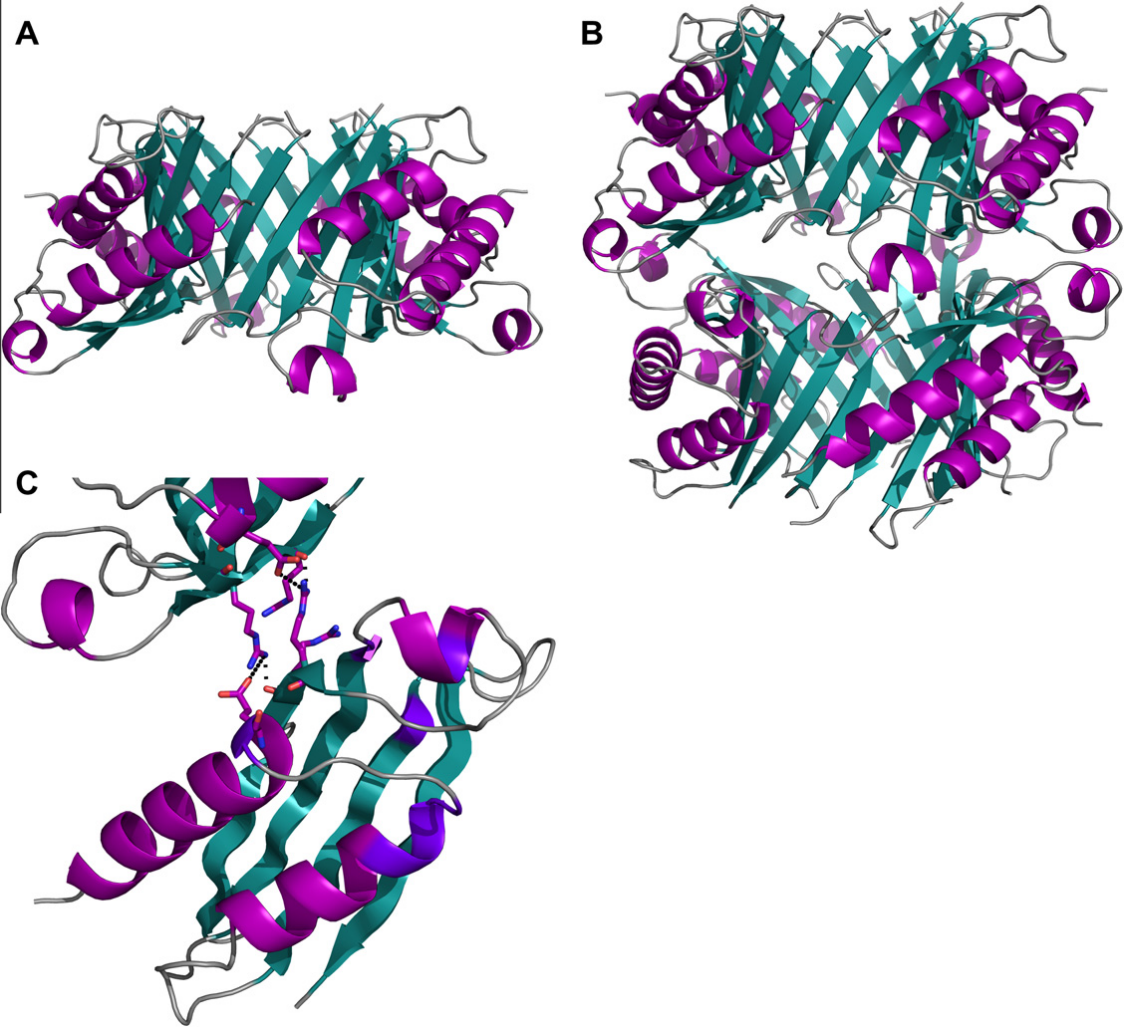
Oligomerisation of FolX. (A) The tetramer ring formed by FolX dimerises to form an octamer (B). (C) The interface between the tetrameric rings is stabilised by four hydrogen bonds (shown as dashed lines) between residues R19, R17 and E72 (shown in sticks). Active site residues are highlighted in purple.

FolX crystallised in space group *I*432, with a single chain in the asymmetric unit. The Protein Interfaces, Surfaces and Assemblies (PDBePISA) server [24] suggests that the oligomeric state, based on the crystallographic symmetry, is tetrameric (Fig. 3A), consisting of a circle made up of the convex sides of the sheets of the subunits facing each other. The interfaces between the subunits are made up by strands β4 on one subunit and β1 on the next in the circle. The contacts comprise 20 residues on each strand, accounting for ∼18% of the total solvent accessible area of the tetramer. The interface is made up by hydrogen bonds formed between a number of residues, mostly involving main chain nitrogen and carboxyl groups (Supplementary Table 1).

Previous studies have suggested that FolX is an octamer [10,25]. However, when the crystal structure is analysed by PDBePISA, it is predicted to be a tetramer. The only interactions between the tetramers, in what would be a dimer of tetramers (Fig. 3B), involve 7% of the solvent accessible area compared with 18% involved in stabilising the tetramer itself. Analysis of the interface between the tetrameric rings, using PDBePISA, indicates that there are 16 hydrogen bonds and 8 salt bridges connecting the two tetramers (Supplementary Table 2), which may suggest that this interaction is significant despite the relatively small interface area.

**Fig. 4:**
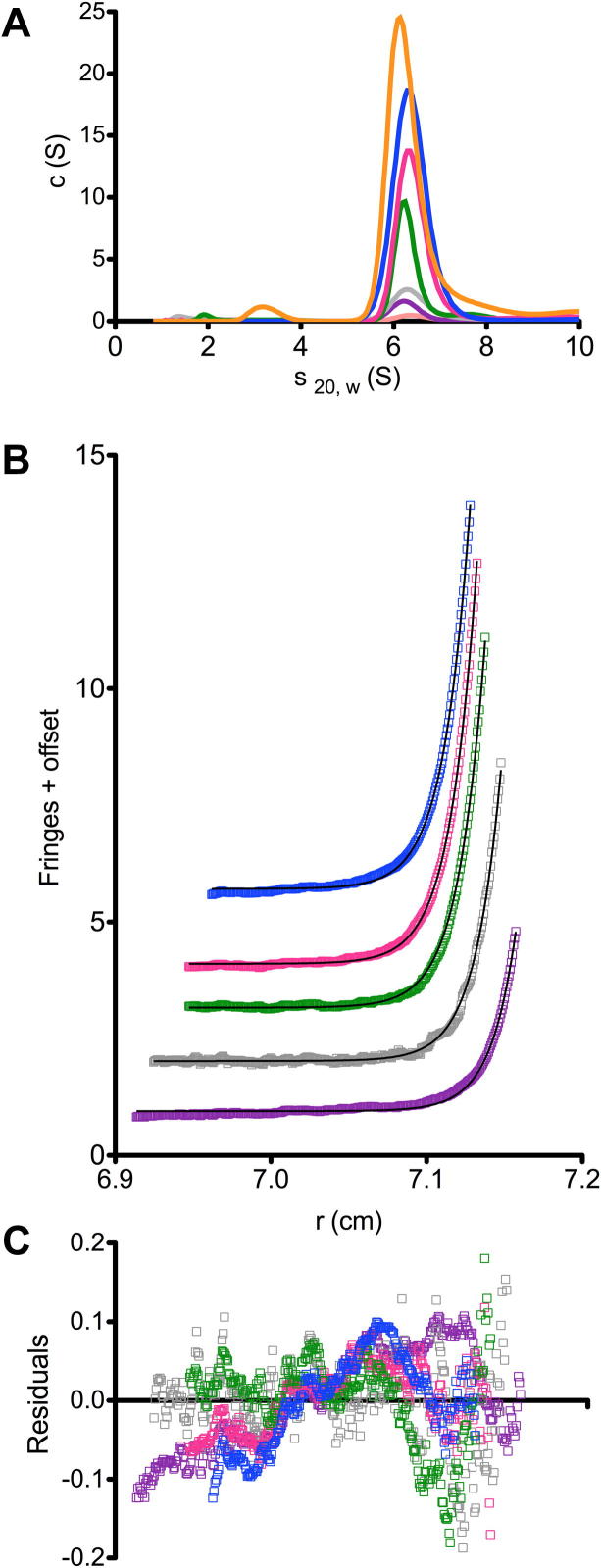
AUC analysis of FolX. (A) c(S) distributions derived via SEDFIT from SV data for varying concentrations of FolX are dominated by a peak at *s*_20,w_ ≈ 4 S. Different concentrations are shown in different colours: 0.2 mg ml^−1^, light pink; 0.5 mg ml^−1^, purple; 1 mg ml^−1^, grey; 2.5 mg ml^−1^, green; 5 mg ml^−1^, pink, 7.5 mg ml^−1^, blue; 10 mg ml^−1^, orange. (B and C) The global fit to SE data using a non-ideal tetramer–octamer self-association model. The experimental data and fits are shown in (B); the residuals for each fit are plotted in (C). Different protein concentrations are represented by the colour scheme used in (A).

In order to try to get conclusive evidence for the quaternary state of FolX, analytical ultracentrifugation was performed. Sedimentation velocity experiments revealed that FolX was present as a single species in solution, as evidenced by a single peak in the concentration distribution of the apparent *s*_20,w_ (c(s), Fig. 4A). The infinite dilution sedimentation coefficient ($s_{20\text{,w}}^{0}$) of FolX, derived from the concentration dependence of *s*_20,w_, determined by fitting the data with a non-interacting discrete species model, is 6.09 ± 0.03 S. This corresponds with the value of $s_{20\text{,w}}^{0}$ computed (using SOMO [26]) for the octamer crystal structure (5.97 S) and not with that computed for the tetramer (3.62 S). Sedimentation equilibrium data fitted with a single species model indicated the presence of a species with a mass of 141,500 ± 6659 Da at infinite dilution (M^0^). This value is slightly lower than the calculated octamer mass of 143,864 Da, therefore, in order to improve the fit of the model parameters, the effects of non-ideality and the presence of a tetramer–octamer equilibrium were introduced into the data analysis. Addition of non-ideality improved the *χ*^2^ of the global fit from 0.01890 to 0.00316. Extending the model to include a tetramer–octamer equilibrium further improved the fit to a *χ*^2^ of 0.00297 and gave a *K*_d_ of 0.887 μM. The fit to the data along with the resultant residuals is shown in Fig. 4B and C.

### 3.3 Active site

Ploom et al. [10] suggested a putative active site for FolX from *E. coli* involving residues that are mostly conserved between *E. coli* and *P. aeruginosa* with only 2 substitutions (Asn to Glu and Lys to Arg, respectively) (Fig. 2B). Most of these residues are disordered and not present in the electron density, implying that the active site of *P. aeruginosa* FolX is flexible in the absence of substrate, or exhibits multiple conformations. The active site does not appear to be affected by the formation of the octamer (Fig. 3C).

### 3.4 SAXS structure

**Fig. 5:**
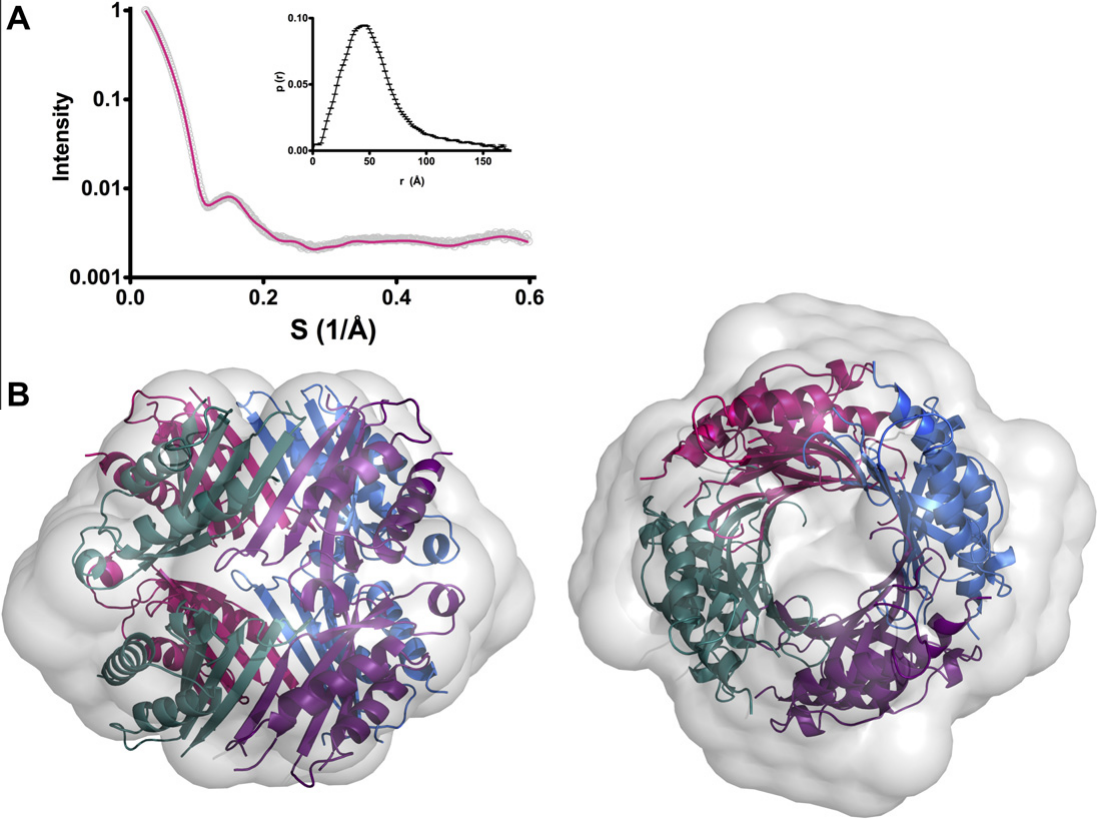
Solution structure of FolX. (A) The experimental scattering data (grey) with the fit of the DAMMIF model to the data shown in pink. Inset is the pairwise distribution p(r) function of the data. (B) The octamer crystal structure superimposed onto the DAMMIF model shown as side and top view.

To further confirm the oligomeric state of FolX, the solution structure was determined using SAXS. Indirect Fourier transformation of the data using GNOM [17] indicated a *D*_max_ of 177 Å and an Rg of 44.11 ± 0.6 Å. An *ab initio* model of FolX was generated using DAMMIF [18], imposing *P*4 symmetry, based on the crystal structure, and the fit of the final averaged model to the experimental data is shown in Fig. 5A. We have determined the *K*_d_ of FolX to be 0.887 μM, therefore at the protein concentration used in the SAXS study 99.5% (by mass) of the protein would have been fully octamerised. The crystal structure of the octamer was superposed onto the 10.5 Å resolution envelope of FolX in solution, (Fig. 5B). The octamer crystal structure fits well into the envelope confirming that this is the true solution oligomeric state.

Here we present the structure of FolX from *P. aeruginosa*, which is an octamer in both the crystal and in solution (in equilibrium with its tetrameric form). Despite only 7% of the surface area of the two tetramers being involved in the interface these interactions are stabilised by 16 hydrogen bonds and 8 salt bridges. Consequently the association of the two tetrameric rings is strong, with a *K*_d_ of 0.887 μM indicating a high affinity association.

The active site of FolX is predicted to comprise residues from two adjacent subunits, which suggests that the tetramer is essential for the activity of the enzyme. The functional need for an octameric form is not apparent from the previously published biochemical data. We speculate that the formation of the octamer may play a role in the stability of FolX, which could be tested by examining the half-life of the protein.

## Figures and Tables

**Table 1:** Data collection, refinement and model quality statistics for FolX. Values in brackets represent the values of the highest resolution shell. R.m.s.d. is root-mean square deviation.

PDB code	4AEY
Space group	*I*432
Unit Cell (Å)	*a* = 133.98
Resolution (Å)	94.74–3.00 (3.16–3.00)
Observed reflections	103 928
Unique reflections	4383
Multiplicity	23.7 (25.1)
Completeness (%)	100.0 (100.0)
*R*_meas_ (%)	5.1 (141.6)
*R*_pim_ (%)	1.1 (38.4)
*I*/*σI*	34.3 (2.9)
Wilson B (Å^2^)	115.3

Protein residues/atoms	107/754
Water molecules	8
*R*_work_ (%)	28.3
*R*_free_ (%)	31.1
R.m.s.d. for bond lengths (Å)/angles (°)	0.01/1.49
Molprobity score	13.9 [97th percentile]
