# Supplementary material for: FolX from Pseudomonas aeruginosa is octameric in both crystal and solution
Source: FEBS Lett. 2012 Apr 24;586(8):1160–5. doi: 10.1016/j.febslet.2012.03.031 (PMC3405516; doi:10.1016/j.febslet.2012.03.031)
Supplement: Supplementary data 1 [file mmc1.doc]

Supplementary Table 1: Hydrogen bonding involved in the interfaces of tetrameric FolX, as determined by PISA.

| Subunit A | Distance (Å) | Subunit B |
| --- | --- | --- |
| Glu113 (N) | 3.80 | Asp15 (O) |
| Glu113 (N) | 2.75 | Lys14 (O) |
| Ser 114 (N) | 3.63 | Lys14 (O) |
| Ser116 (N) | 3.06 | Arg12 (O) |
| Thr118 (N) | 2.82 | Arg10 (O) |
| Ala120 (N) | 3.11 | Met8 (O) |
| Thr118 (O) | 3.22 | Arg10 (N) |
| Ser116 (O) | 3.03 | Arg12 (N) |
| Ser114 (O) | 3.02 | Lys14 (N) |
| Phe111 (O) | 3.18 | Arg17 (N) |
