# Supplementary material for: FolX from Pseudomonas aeruginosa is octameric in both crystal and solution
Source: FEBS Lett. 2012 Apr 24;586(8):1160–5. doi: 10.1016/j.febslet.2012.03.031 (PMC3405516; doi:10.1016/j.febslet.2012.03.031)
Supplement: Supplementary data 2 [file mmc2.doc]

Supplementary Table 2: Hydrogen bonding and salt bridges involved in the interfaces of octameric FolX, as determined by PISA.

| Tetramer A | Distance (Å) | Tetramer B |
| --- | --- | --- |
| *Hydrogen bonds* |  |  |
| Arg19 (NH2) | 2.58 | Arg17 (O) |
| Arg19 (NH1) | 3.42 | Glu72 (OE2) |
| Arg17 (O) | 2.58 | Arg19 (NH2) |
| Glu72 (OE2) | 3.42 | Arg219 (NH1) |
| *Salt bridges* |  |  |
| Arg19 (NH1) | 3.42 | Glu72 (OE2) |
| Glu72 (OE2) | 3.42 | Arg219 (NH1) |
